# Age Increases Monocyte Adhesion on Collagen

**DOI:** 10.1038/srep46532

**Published:** 2017-05-17

**Authors:** Samira Khalaji, Lisa Zondler, Fenneke KleinJan, Ulla Nolte, Medhanie A. Mulaw, Karin M. Danzer, Jochen H. Weishaupt, Kay-E. Gottschalk

**Affiliations:** 1 Institute for Experimental Physics, Ulm University, Ulm, Germany; 2 Department of Neurology, Ulm University, Ulm, Germany; 3 Institute for Experimental Cancer Research, Ulm University, Ulm, Germany

## Abstract

Adhesion of monocytes to micro-injuries on arterial walls is an important early step in the occurrence and development of degenerative atherosclerotic lesions. At these injuries, collagen is exposed to the blood stream. We are interested whether age influences monocyte adhesion to collagen under flow, and hence influences the susceptibility to arteriosclerotic lesions. Therefore, we studied adhesion and rolling of human peripheral blood monocytes from old and young individuals on collagen type I coated surface under shear flow. We find that firm adhesion of monocytes to collagen type I is elevated in old individuals. Pre-stimulation by lipopolysaccharide increases the firm adhesion of monocytes homogeneously in older individuals, but heterogeneously in young individuals. Blocking integrin α_x_ showed that adhesion of monocytes to collagen type I is specific to the main collagen binding integrin α_x_β_2_. Surprisingly, we find no significant age-dependent difference in gene expression of integrin α_x_ or integrin β_2_. However, if all integrins are activated from the outside, no differences exist between the age groups. Altered integrin activation therefore causes the increased adhesion. Our results show that the basal increase in integrin activation in monocytes from old individuals increases monocyte adhesion to collagen and therefore the risk for arteriosclerotic plaques.

Monocytes represent approximately 5–10% of human peripheral blood leukocytes. These cells originate from myeloid precursor cells in bone marrow and later circulate in the blood, bone marrow and spleen and finally enter the tissues^1^. Monocytes play a vital role for innate and adaptive immune responses to pathogens and employ most of their functions outside of the vascular compartment; therefore, trafficking and migration processes are essential for these cells^2^. Recruitment of blood monocytes to the site of infection and injury is described to be influenced by the inflammatory milieu (e.g. adhesion molecules, chemokines and pathogen-associated pattern-recognition receptors)^1^,^3^.

Extensive studies have been conducted on circulating monocytes and their interaction with endothelium associated with age and age-related diseases such as atherosclerosis. For example, former studies documented the altered expression of adhesion molecules such as L-selectin (CD62L) and α_M_β_2_ (CD11b/CD18 or MAC1) on monocytes from older individuals and hypothesized subsequent impaired function of monocytes^1^,^3^. Furthermore, in different experimental systems, including *in vitro* and *in vivo* studies, it was demonstrated that atherogenic conditions (e.g. hypercholesterolemia) potentially stimulate adhesion of monocytes to the endothelial cells^4^. However, despite the fact that the migratory function of monocytes through endothelium seems to be important in both healthy aging and age-related diseases such as atherosclerosis, no studies were performed specifically to understand if the rolling and adhesion of monocytes on collagen are affected by aging. During invasion of monocytes into the arterial walls at the site of micro-injuries, these cells interact with extracellular matrix components, especially with collagen type I, a major element of normal arterial wall matrix and atherosclerotic plaques^5^,^6^. Therefore, we decided to scrutinize the interaction of monocytes with collagen in an age-dependent fashion.

In this study, we investigated whether age affects function of rolling and adhesion of monocytes under flow on collagen type I in a flow chamber assay. Since rolling and adhesion are enhanced on inflamed venules^7^, we tested rolling and adhesion of monocytes also under the influence of Lipopolysaccharide (LPS). LPS is a major component of the outer surface of gram negative bacteria that is recognized by monocytes^8^. Human monocytes are strongly sensitive to LPS and respond by expressing different inflammatory cytokines such as tumour necrosis factor-alpha (TNFα)^9^. In the current study, we found that adhesion of monocytes on collagen type I is increased by age. Further our results show that LPS pre-stimulated monocytes adhere on average 30% more often in both age groups. However, monocytes from older individuals respond to LPS homogenously in contrast to monocytes from young individuals, where strong individual-to-individual differences are observed. Rolling of monocytes seems to be unaffected by age and LPS stimulation. Since monocyte adhesion is strongly mediated by integrins, we went on to test integrin expression. However, neither we nor others^10^ found an increase in the expression of integrins specific for collagen type I. Therefore, the observed increase in adhesion of monocytes from older individuals is either integrin-independent or caused by altered signalling pathways leading to an increase in integrin activation.

## Results

### Monocytes’ rolling and rolling velocity is not affected by age

**Figure 1:**
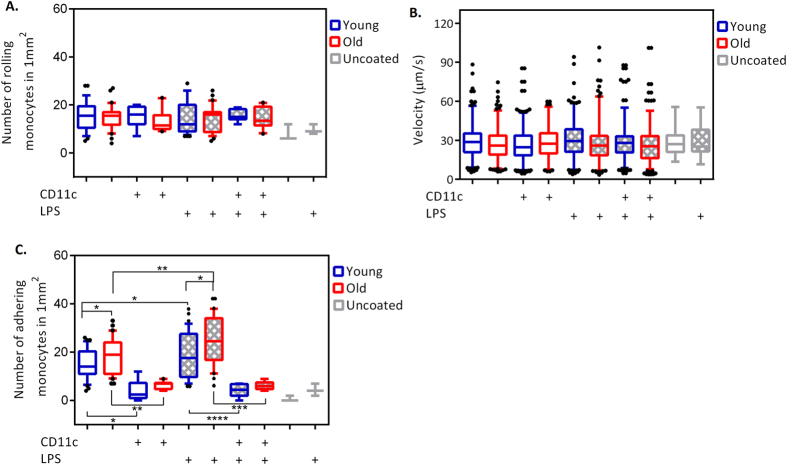
Rolling, adhesion and rolling velocity of monocytes. (**A**) The number of rolling monocytes in 1 mm^2^. (**B**) Monocyte rolling velocity data for both young and old, as well as LPS pre-stimulated samples. (**C**) The number of firmly adhering monocytes in 1 mm^2^. The unpaired Student’s t test is used to determine the statistical difference between the groups. The central mark is the median, the edges of the box are the 25^th^ and 75^th^ percentiles, the whiskers are extending to the to the 10th percentile and up to the 90^th^, and points below and above the whiskers are shown as black dots. For all the experiments, for young group, n = 16 and for old group, n = 14. For CD11c blocking condition, for young group, n = 4 and for old group, n = 5.

Monocytes recruitment is believed to follow the general leukocytes trafficking and adhesion paradigm which involves rolling, adhesion and transmigration through endothelial cells^11^. Consequently, the initial step for firm adhesion is the rolling process^12^. To assess the rolling behaviour of monocytes in different individuals based on age, we counted the number of monocytes which rolled on the collagen type I substrate of micro-fluidic chamber. Our result show that the number of rolling monocyte is unaffected by age in different individuals (Fig. 1A). The average number of rolling monocytes is 16 ± 2 and 15 ± 1 in 1 mm^2^ in old and young individuals, respectively.

The number of rolling monocytes in pre-stimulated samples with LPS shows no significant difference neither by age nor compared to control (un-treated) samples. The average number of rolling monocytes is 15 ± 2 and 14 ± 2 in 1 mm^2^ in LPS pre-stimulated samples for young and old groups, respectively (Fig. 1A).

Generally, the efficient conversion of the rolling process to firm adhesion in leukocytes depends on the amount of time one cell spends in close contact with endothelial cells^13^. To determine whether age increases the contact time between monocytes and the collagen type I substrate during rolling, we measured the rolling velocity in both of the experimental groups after 10 minutes of constant shear stress in non- and pre-stimulated monocytes (Fig. 1B).

Rolling monocytes from young and old individuals had an average velocity of 28.7 ± 0.5 μm/s and 27 ± 0.5 μm/s, respectively. To determine if the rolling velocity is affected by both age and inflammatory condition, we also determined the rolling monocyte velocity in pre-stimulated samples with LPS. Pre-stimulated monocytes from young and old individuals rolled at an average velocity of 30.3 ± 0.6 and 27 ± 0.6 μm/s, respectively.

### Monocytes from old individuals adhere more frequently

In our flow chamber assay a significant higher number of firmly adhering monocytes with an average of 19 ± 2 in 1 mm^2^ from old donors is found compared to the monocytes from young donors with an average of 15 ± 2 in 1 mm^2^ (P < 0.05) (Fig. 1C).

To investigate the impact of bacterial LPS on monocytes adhesion, we proceed with pre-stimulation of monocytes with LPS prior to flowing the cells through the chamber. LPS interacts with CD14 and makes a complex with surface toll like receptor (TLR)4. This complex activates signalling pathways that subsequently activates integrins which play a critical role in the adhesion cascade^14^. We observed that the number of firmly adhering monocytes increased significantly in both monocytes from the young group with an average of 19 ± 2 in 1 mm^2^ (P < 0.05), and from the old group with an average of 26 ± 1 in 1 mm^2^ (P ≤ 0.01) compared to the number of firmly adhering monocytes in young and old groups without LPS pre-stimulation, respectively.

Additionally, our data show significantly higher number of firmly adhering monocytes in LPS pre-stimulated monocytes from old individuals compared to LPS pre-stimulated monocytes from young individuals (P < 0.05) (Fig. 1C). In the absence of collagen type I, hardly any monocytes adhere (Fig. 1C).

### Monocyte adhesion is mediated by Integrin α_X_β_2_ (CD11c/CD18 or gp150-95)

To study the specific role of CD11c/CD18 in adhesion of monocytes to collagen type I under flow, we blocked CD11c by a ligand binding site specific monoclonal antibody (mAB). The number of firmly adhering cells to collagen type I under flow was decreased significantly back to the background level in the absence of collagen in non (P < 0.05) and LPS pre-stimulated (P < 0.01) monocytes from young individuals as well as in non and LPS pre-stimulated (P ≤ 0.0001) monocytes from old individuals after using mAB against CD11c (Fig. 1C).

Blocking of CD11c by ligand binding site specific mAB did not interfere either with the number of rolling monocytes, or with the velocity of cells on collagen type I under flow (Fig. 1A,B).

### Expression of α_X_β_2_ integrin on monocytes is not altered by age

**Figure 2:**
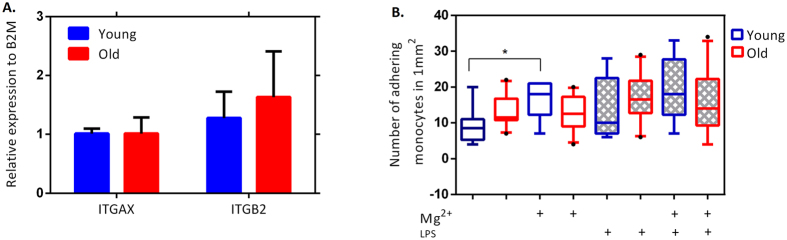
Relative gene expression of α_X_β_2_ integrin and monocytes integrins activation. (**A**) The gene expression of α_X_β_2_ (ITGAX and ITGB2) are normalized relatively to the expression of B2M on monocytes. For both old and young groups, n = 5 and non-parametric Mann–Whitney U test is used to determine the statistical difference between the groups. Error bars represent SEM. (**B**) The number of firmly adhering monocytes in 1 mm^2^. The non-parametric Mann–Whitney U test is used to determine the statistical difference between the groups. After Mg^2+^ stimulation, no differences exist between the age groups, showing equal amounts of adhesion receptors on the monocyte surface. For young group, n = 4 and for old group, n = 5.

Since integrin α_X_β_2_ is a responsible integrin to bind and interact with ECM molecules including collagen type I^15^, we performed qRT-PCR to assess expression of the two subunits of α_X_ (ITGAX) and β_2_ (ITGB2) in monocytes from individuals with different ages. Our data show that the expression of both genes is not affected by age on mRNA level (Fig. 2A).

### Integrin activation by divalent cation Mg^2+^ increases adhesion of monocytes only in young individuals

Furthermore, monocyte adhesion triggered by collagen type I was studied in the presence of high concentration of divalent cation of 5 mM Mg^2+^. Under these conditions, all the integrins become un-specifically activated from the outside without the requirement of cellular signalling cascades^5^,^16^. Only monocytes from young individuals, which are not pre-stimulated with LPS, adhere significantly more often under these conditions (Fig. 2B). Interestingly, there is no statistically significant difference between monocytes from young and old individuals after Mg^2+^ stimulation in both non and LPS pre-stimulated monocytes (Fig. 2B), showing that the number of surface receptors is not different between the two age groups.

### Monocytes from older people are homogeneously activated by LPS in contrast to monocytes from young individuals

**Figure 3:**
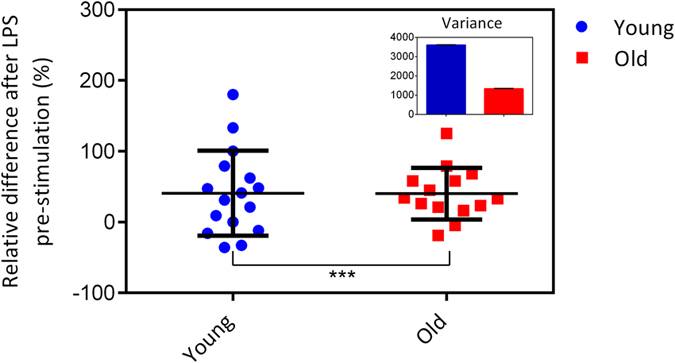
LPS pre-stimulation of monocytes. The response of monocytes to LPS pre-stimulation based on the relative number of firmly adhering monocytes in LPS pre-stimulated monocytes to non-treated monocytes. Each dot represents data for one individual. The central mark is the average and the error bars are SD. For all the experiments, for young group n = 16 and for old group n = 14 individuals. The Two-way ANOVA test is used to determine if the monocytes adhesion is affected significantly by LPS pre-stimulation and age. The small box represents the variance for both of the data set.

**Figure 4:**
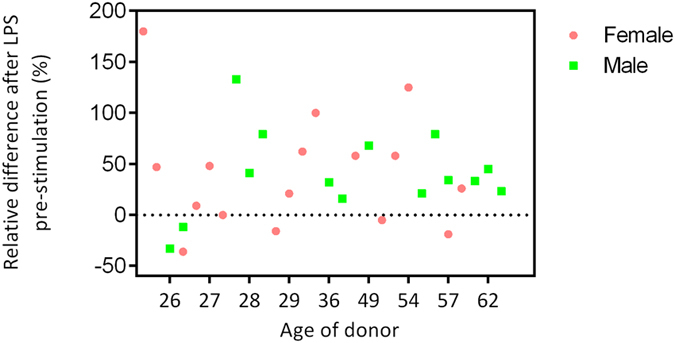
Monocytes response to LPS pre-stimulation based on the age and gender of donors. Each dot represents data for one individual and is based on the relative number of firmly adhering monocytes in LPS pre-stimulated monocytes to non-treated monocytes in one individual.

Age-associated enhanced susceptibility to infection have been documented in older individuals compared to young adults^17^. Hence, the capacity to become activated by LPS may alter with age. Analysing in greater details, the age-related differences of monocyte adhesion revealed that most of the individuals from the old group (12 out of 14) had an increase in firm adhesion of monocytes after LPS pre-stimulation. Interestingly, the monocytes from younger individuals displayed a very heterogeneous response to LPS pre-stimulation. Monocytes from 4 young individuals showed a reduction and one person with almost no changes in adhesion after pre-stimulation, while adhesion of monocytes from 11 young individuals increased after LPS pre-stimulation. The trend of enhanced adhesion after LPS pre-stimulation is dominant in older individuals (Fig. 3). Using Two-way ANOVA test, we confirmed that the adhesion of monocytes is significantly affected by both LPS pre-stimulation and age (P ≤ 0.00 1). No effect of gender is observable (Fig. 4).

## Discussion

Monocytes contribute to immune surveillance and the inflammatory responses in inflammatory diseases such as atherosclerosis. Monocyte recruitment and trafficking across the blood vessels walls to the sites of injury requires a series of events including slow rolling, firm adhesion and transmigration through the endothelium^2^. Selectins such as L-selectin mediate the primary tethering and rolling of monocytes and integrin receptors including α_M_β_2_, α_X_β_2_ and VLA-4 (CD49d/CD29) mediate firm monocyte adhesion^18^,^19^. Peripheral blood monocytes are additionally able to adhere to ECM proteins in particular collagen type I, which is a major element of the normal arterial wall matrix and atherosclerotic plaques^6^. The ECM is exposed to the monocytes at the sites of micro-injuries. Furthermore, the collagen content of vessel walls has been shown to be increased in older animal models and human compared to younger animals and human^20^. This can be highly significant in some of the age-associated diseases like atherosclerosis, in particular as 60% of the proteins in a plaque is made of collagens^21^.

In contrast to adhesion of monocytes to endothelium^18^, little is known about interaction of these cells with ECM proteins. In fact, our study is the first study investigating the influence of aging on human peripheral blood monocytes firm adhesion and rolling under physiological flow on a major ECM composition of blood vessel walls, collagen type I. However, monocytes are in close interaction with collagen type I during invasion to inflamed tissue or at vascular injuries^22^. Therefore, our set up is a suitable model for vascular injuries and atherosclerotic lesions.

Our study shows that the adhesion of peripheral blood monocytes to collagen type I increases with age. The major integrin receptors for collagen type I on monocytes are α_1_β_1_, α_2_β_1_ and α_X_β_2_^5^,^23^. Previously, it has been demonstrated that both α_1_β_1_ and α_2_β_1_ expression is only moderate on monocytes^1^ and we have shown that their expression is not changed by age^10^. Blocking integrin α_X_ with a ligand binding site specific mAB showed that adhesion of monocytes on collagen type I was dependent on this integrin. The observed increase in adhesion in monocytes from older individuals could therefore be caused by increased expression of integrin α_X_. Our results presented here, however, show no age-dependent changes in the expression of the receptor for collagen type I α_x_β_2_ integrin on mRNA level on ”classical” CD14++ monocytes. To further test whether the number of integrin-adhesion receptors is responsible for the increase in adhesion in monocytes from older individuals, we activated all the integrins on the monocytes’ surface with 5 mM Mg^2+^. Our data show a significant increase in adhesion for monocytes only from young individuals. After Mg^2+^ stimulation, there is no difference in the adhesion between the age groups. This strongly corroborates that the number of expressed integrins on the monocyte surface does not cause the differences in adhesion between the age group.

Since integrin expression is not the cause for the increase in adhesion, increased activation of expressed integrins may cause the observed effects. Integrins are often activated upon encounter of chemokines. A major chemokine involved in monocyte adhesion is the monocyte chemoattractant protein-1 (MCP-1 or CCL2). MCP-1 plays pivotal roles in monocytes recruitment^24^ and thus in the development of atherosclerosis^25^. Indeed, MCP-1 triggers firm adhesion of monocytes to vascular endothelium under flow conditions^26^. The study by Seidler, *et al*. reported an increase of systemic serum concentration of MCP-1 by age^1^. Further, interaction of discoidin domain receptor 1 (DDR1b), a non-integrin, non-adhesive collagen receptor, with collagen is shown to up-regulate the secretion of MCP-1 from human monocyte-derived macrophages^27^. Interaction of MCP-1 with its receptor activates the collagen-binding β_1_ and β_2_ integrins on monocytes^28^ and with this, increase adhesion. Therefore, our results of an age-dependent increases in firm adhesion of monocytes on collagen type I may point towards the following mechanism: monocytes from older individuals have a heightened capacity to produce MCP-1 and upon activation of these cells via interaction of DDR1b with collagen, monocytes of aged people produce and release larger amount of MCP-1.

An important process in inflammatory response against gram-negative pathogens is the recruitment of monocytes into the affected tissue. Therefore, investigation of the effect of age on monocyte function activated by bacterial products (e.g. LPS) is crucial^29^. The receptor required for LPS signal transduction is TLR4. The majority of previous studies showed an age-related reduced expression^30^or no changes^31^ of TLR4 on human classical monocytes. In particular, our results show that LPS pre-stimulation leads to an increase in the inflammatory response of monocytes as the number of firm adhering monocytes increase in LPS pre-stimulated samples compared to control (non-treated) samples. This increase is significant in monocytes from both young and old group. However, LPS pre-stimulation leads to a very homogeneous response in monocytes from older individuals, but to a very heterogeneous response in monocytes from younger individuals. Indeed, if the young individuals show an increase in adhesion, this increase is at least as strong as for the older individuals. The heterogeneity of this response renders a statistical evaluation of the response difficult. Interestingly, on average the response to LPS is identical in both age groups: on average, LPS increases adhesion by approximately 30%, independent of age. This is in accord with the previous studies about the LPS receptor^31^.

Taken together, our study shows that aging is associated with significant increase in firm adhesion of monocytes on collagen type I and a homogenization of the response of monocytes to LPS pre-stimulation. This strongly suggests that interactions between monocytes and collagen type I that are involved in the series of events that leads to age-associated diseases like atherosclerosis, are increased with age. Future studies regarding adhesion molecules and downstream mechanism underlying the increased adhesion by age are promising to unravel the complex events leading to atherosclerosis.

## Methods and Material

### Ethical approval and blood donors

All human sampling and procedures were performed and approved in accordance with ethical standards of the institutional research committee (Ethics Committee of Ulm University) and based on the 1964 declaration of Helsinki and later amendments or comparable ethical standards. Informed consent was obtained from all subjects to participate in our study. All healthy young and old donors were recruited at Ulm University and Ulm University clinic. 16 healthy young donors (range 24–36 years old, median age 27.5, 10 females and 6 males), and 14 healthy old donors (range 44–73 years old, median age 56, 6 females and 8 males) were chosen for our experiments (Supplementary Table 1). Blood samples from individual with acute or chronic condition that might affect the immune system were excluded.

### Isolation of primary human monocytes

Peripheral blood monocyte cells were isolated by centrifugation of the venous whole blood (30 min, 500 × g, room temperature and without brakes) on an Histopaque^®^ density gradient (Sigma, MO, USA, #10771).

After washing (2×) with PBS and MACS™ buffer (75 mM BSA + 2 mM EDTA in PBS), a positive selection of CD14^++^ cells were performed using magnetic beads conjugated with monoclonal anti-human CD14 antibodies (Miltenyi Biotec, Germany, #130-050-201).

Monocytes were cultured with density of 1 × 10^6^ per ml in RPMI 1640 medium (Life Technologies, CA, USA, #31870-025) supplemented with 1% Penicillin or Streptomycin (PAA, Austria, #P11-010). Cells were maintained at 37 °C and 5% CO_2_.

To pre-stimulate monocytes, LPS (Sigma, MO, USA, #L4391) was added to the CD14^++^ monocytes culture (final concentration 100 ng/ml) 24 h before the measurements. Subsequently, the cells were washed with PBS and suspended in fresh RPMI medium (1 × 10^6^/ml).

### Blocking of CD11c using mAb on monocytes

LEAF™ Purified anti-human CD11c antibody (monoclonal IgG1 clone 3.9) (Biolegend, CA, USA, #301616) was used to study the role of integrin α_X_β_2_ (CD11c/CD18) on the adhesion and rolling of monocytes on collagen type I. 1 × 10^6^ monocytes were incubated with 10 μg IgG/ml for 60 min at 37 °C. To avoid recycling of unoccupied integrins to the cell surface, and decrease in the efficiency of blocking mAB, unbound antibodies were not washed away^32^. The effect of receptor specific antibody was compared to untreated monocyte samples that were incubated at the same condition without any specific antibody.

### Monocytes integrins activation using divalent cation Mg^2+^

The effect of divalent cation Mg^2+^ on monocyte adhesion and rolling was studied. Monocytes were pre-incubated with RPMI medium with a constant concentration of 5 mM Mg^2+^ for 60 min at 37 °C^5^. The tubes containing the pre-incubated monocytes were stirred occasionally. RPMI solution with 5 mM of Mg^2+^ was maintained during the flow experiment.

### Monocytes adhesion and rolling assay under physiological flow

**Figure 5:**
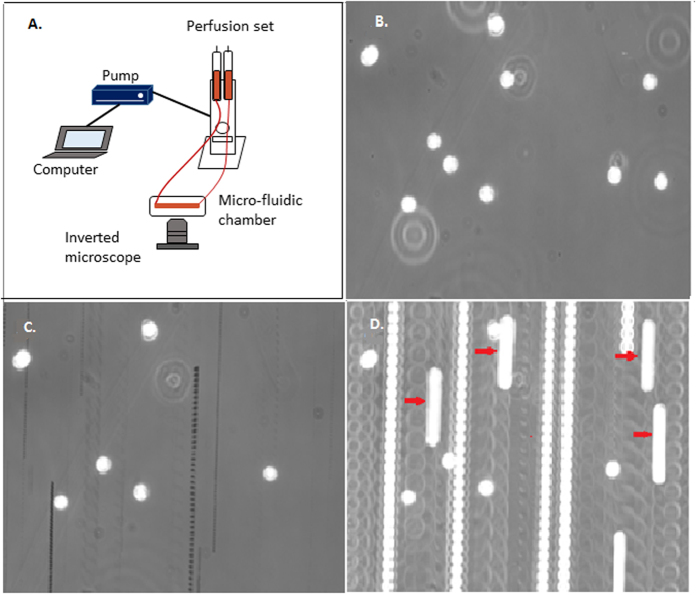
Example images of the flow set up. (**A**) Schematic view of the flow set up. (**B**) Snapshot from one field of view. (**C**) 4 second minimization image showing that 6 of the 10 monocytes visible in (**A**) are firmly adhered to the collagen type I substrate during 4 second. Therfore, 4 monocytes rolled. (**D**) 4 second maximization image which is used to quantify the rolling velocity of monocytes. Only the continues long blurs are considered as rolling monocytes (marked with red arrows).

Physiological flow condition was mimicked *in vitro* using a micro-fluidic chamber (μ-slides, ibidi, Germany, #80191) coated with collagen type I (ibidi, Germany, #50201) and ibidi Pump System (ibidi, Germany, #10902) (Fig. 5A). Samples were diluted to 1 × 10^5^ cells/ml with 9 ml of RPMI medium supplemented with 10% FCS (Bio & sell, Nürnberg, Germany, #FCS.GP.0500) and perfused through the chamber at laminar shear stress of 0.6 dyn/cm^2^ (0.06 Pa, representing the shear rates within capillaries and small venules^14^) for 10 minutes as described previously^33^. Experiments were performed in duplicate/triplicate. The interaction of monocytes and collagen type I substrate was observed and recorded using an inverted light microscope (Axio Observer Microscope A1., Carl Zeiss MicroImaging GmbH, Germany) and a high speed camera (DMK23U618, Imaging Source Europe GmbH, Germany). Images were acquired after 10 minutes of constant flow for 4 seconds with a frequency of 30 frames/s from six different areas (594 × 446 μm) of each chamber. For control experiment (uncoated), monocytes were perfused through non-treated chambers. These chambers absorb FCS (supplemented in RPMI medium) but are not coated by any specific ECM adhesion molecules.

### Image processing and data analysis

Analysis of all images was performed using the public domain *ImageJ* program (available at http://rsb.info.nih.gov/ij/). We define firm adhesion as a monocyte remains stationary for at least 4 seconds as described earlier^33^. Firmly adherent and rolling monocytes are differentiated by acquiring two images for each field (Fig. 5). The first image is a single frame “snapshot” which shows both firmly adherent and rolling monocytes (white circles on the dark background) (Fig. 5B). Monocytes flowing by without contacting the collagen substrate are out of focus and therefor do not appear in this image. The second image is a 4 second minimization image by *ImageJ* (Fig. 5C)^33^. In this image, the pixel intensities from all frames are stored in a single image (minimization image), but only if the intensity of a particular pixel is lower than the previous stored intensity^33^. Since monocytes appear as phase-bright circles on a dark background if a monocyte moves (rolling), the pixel intensity of its previous location are replaced by the dark background (lower intensity). The result is that only monocytes, which firmly adherent during the 4 second of image acquisition time, appear in this image. Subtraction of the number of adhering monocytes from the total number of monocytes at the snapshot is the number of rolling monocytes^33^. Rolling velocity was quantified as described before^33^. In summary, the pixel intensities of 120 frames were maximised in to a final image. The result of this procedure is that rolling monocytes appeared as long continuous blurs (Fig. 5D). The length of each blur (μm) divided by time (s) is the velocity of that monocyte. We analysed only cells with visible start and end points which limits the maximum velocity to approximately 112 μm/s. The velocity of 150–207 monocytes was measured for each condition.

### RNA isolation

RNA isolation was done using the RNeasy Plus kit (QIAGEN, Netherlands, #74134) from approximately 5 × 10^6^ monocytes for each sample according to the manufacturer’s instructions. RNA yield was quantified using a NanoDrop (Thermo Scientific, MA, USA).

### Reverse transcription and quantitative real time PCR

Reverse transcription was performed using 500 ng RNA form each sample using the iScript^TM^ cDNA Synthesis Kit (Bio-Rad, CA, USA, #170-8890) according to the producer’s instructions. The cDNA was diluted and 10 ng of cDNA was used for the qRT-PCR. qRT-PCR was performed using the iQ™ SYBR^®^ Green Supermix (Bio-Rad, CA, USA #170-8880) on the CFX96 Real-Time System (Bio-Rad). The specific sequencing primers were designed using primerblast (http://www.ncbi.nlm.nih.gov/tools/primer-blast/) to quantify expression of ITGAX (forward: GCTCCATTGGGGGTCTGTTG and reverse: GAGGGTAATGGGGAGTGGG, annealing at 62 °C) and ITGB2 (forward: CGCCTCCAGCTGAGGTTT and reverse: ACTCCTGAGAGAGGACGCAC, annealing at 62 °C) (Biomers, Germany). The gene expression was normalized relatively to Beta-2-Microglobulin (B2M) cDNA using the 2^−ΔΔCt^ method^34^.

### Statistical analysis

The *GraphPad PRISM 6* software was employed to perform statistical analysis of data and to create figures. Data are represented as mean ± SEM if not mentioned otherwise. For all the data set, column statistics was performed to evaluate the normality distribution. If all the values were normally distributed based on the Gaussian distribution, the unpaired Student’s t test was used to estimate the statistical difference between different groups and treatments. Otherwise, the non-parametric Mann–Whitney U test was applied. The two-way ANOVA test is used to determine if adhesion of monocytes is affected significantly by both LPS treatment and age factors. Note that P > 0.05 is not significant and display of symbols *, **, *** and **** means P is ≤0.05, ≤0.01, ≤0.001 and ≤0.0001, respectively.

## Additional Information

**How to cite this article:** Khalaji, S. *et al*. Age Increases Monocyte Adhesion on Collagen. *Sci. Rep.* **7**, 46532; doi: 10.1038/srep46532 (2017).

**Publisher's note:** Springer Nature remains neutral with regard to jurisdictional claims in published maps and institutional affiliations.

## Supplementary Material

Supplementary Table 1
