# Supplementary material for: Age Increases Monocyte Adhesion on Collagen
Source: Sci Rep. 2017 May 17;7:46532. doi: 10.1038/srep46532 (PMC5434452; doi:10.1038/srep46532)
Supplement: Supplementary Table 1 [file srep46532-s1.pdf]

Age Increases Monocyte Adhesion on Collagen.

Samira Khalaji[1], Lisa Zondler[2+], Fenneke KleinJan[1+], Ulla Nolte[1], Medhanie A. Mulaw[3], Karin M. Danzer[2], Jochen H. Weishaupt[2], and Kay-E. Gottschalk[1*]

[1] Institute for Experimental Physics, Ulm University, Ulm, Germany

[2] Department of Neurology, Ulm University, Ulm, Germany

[3] Institute for Experimental Cancer Research, Ulm University, Ulm, Germany

[*]kay.gottschalk@uni-ulm.de

[+]These authors contributed equally to this work.

A.

Sample	Age	Sex
1	24	f
2	25	f
3	26	m
4	27	f
5	27	f
6	27	f
7	27	f
8	27	m
9	28	m
10	28	m
11	28	m
12	29	f
13	29	f
14	30	f
15	31	f
16	36	m

B.

Sample	Age	Sex
1	44	m
2	48	f
3	49	m
4	50	f
5	53	f
6	54	f
7	56	m
8	56	m
9	57	m
10	57	f
11	59	f
12	62	m
13	62	m
14	73	m

Supplementary Table 1. The summary of the main characteristics of enrolled subjects. A. young blood donors. B. Old blood donor.
